# Validation of a Personalized Hearing Screening Mobile Health Application for Persons with Moderate Hearing Impairment

**DOI:** 10.3390/jpm11101035

**Published:** 2021-10-16

**Authors:** Lok-Yee Joyce Li, Shin-Yi Wang, Jinn-Moon Yang, Chih-Jou Chen, Cheng-Yu Tsai, Lucas Yee-Yan Wu, Te-Fang Wu, Cheng-Jung Wu

**Affiliations:** 1 Department of Medicine, Shin Kong Wu-Ho-Su Memorial Hospital, Taipei 111, Taiwan; b101102173@tmu.edu.tw; 2 School of Medicine, College of Medicine, Taipei Medical University, Taipei 110, Taiwan; wulucasyeeyan@gmail.com; 3 Department of Otolaryngology, School of Medicine, College of Medicine, Taipei Medical University, Taipei 110, Taiwan; 4 Department of Otolaryngology, Shuang Ho Hospital, Taipei Medical University, New Taipei 23561, Taiwan; 5 Department of Nursing, National Taiwan University Hospital, Hsinchu Branch, Hsinchu 300, Taiwan; G80733@hch.gov.tw; 6 Institute of Bioinformatics and Systems Biology, National Yang Ming Chiao Tung University, Hsinchu 300, Taiwan; moon@faculty.nctu.edu.tw; 7 Department of Biological Science and Technology, National Chiao Tung University, Hsinchu 300, Taiwan; 8 Master Program in School of Nursing, Taipei Medical University, Taipei 110, Taiwan; m432110018@tmu.edu.tw; 9 Department of Civil and Environmental Engineering, Imperial College London, London SW7 2BT, UK; ct619@ic.ac.uk; 10 Department of Otolaryngology—Head and Neck Surgery, Kaohsiung Veterans General Hospital, Kaohsiung 813, Taiwan; doctorwu0617@gmail.com; 11 Ph.D. Degree Program in Biomedical Science and Engineering, National Yang Ming Chiao Tung University, Hsinchu 300, Taiwan

**Keywords:** hearing impairment, conductive hearing loss, smartphone, audiometry, mobile health

## Abstract

Hearing impairment is a frequent human sensory impairment. It was estimated that over 50% of those aged >75 years experience hearing impairment in the United States. Several hearing impairment–related factors are detectable through screening; thus, further deterioration can be avoided. Early identification of hearing impairment is the key to effective management. However, hearing screening resources are scarce or inaccessible, underlining the importance of developing user-friendly mobile health care systems for universal hearing screening. Mobile health (mHealth) applications (apps) act as platforms for personalized hearing screening to evaluate an individual’s risk of developing hearing impairment. We aimed to evaluate and compare the accuracy of smartphone-based air conduction and bone conduction audiometry self-tests with that of standard air conduction and bone conduction pure-tone audiometry tests. Moreover, we evaluated the use of smartphone-based air conduction and bone conduction audiometry self-tests in conductive hearing loss diagnosis. We recruited 103 patients (206 ears) from an otology clinic. All patients were aged ≥20 years. Patients who were diagnosed with active otorrhea was excluded. Moderate hearing impairment was defined as hearing loss with mean hearing thresholds >40 dB. All patients underwent four hearing tests performed by a board-certified audiologist: a smartphone-based air conduction audiometry self-test, smartphone-based bone conduction audiometry self-test, standard air-conduction pure-tone audiometry, and standard bone conduction pure-tone audiometry. We compared and analyzed the results of the smartphone-based air conduction and bone conduction audiometry self-tests with those of the standard air conduction and bone conduction pure-tone audiometry tests. The sensitivity of the smartphone-based air conduction audiometry self-test was 0.80 (95% confidence interval CI = 0.71–0.88) and its specificity was 0.84 (95% CI = 0.76–0.90), respectively. The sensitivity of the smartphone-based bone conduction audiometry self-test was 0.64 (95% CI = 0.53–0.75) and its specificity was 0.71 (95% CI = 0.62–0.78). Among all the ears, 24 were diagnosed with conductive hearing loss. The smartphone-based audiometry self-tests correctly diagnosed conductive hearing loss in 17 of those ears. The personalized smartphone-based audiometry self-tests correctly diagnosed hearing loss with high sensitivity and high specificity, and they can be a reliable screening test to rule out moderate hearing impairment among the population. It provided patients with moderate hearing impairment with personalized strategies for symptomatic control and facilitated individual case management for medical practitioners.

## 1. Introduction

Hearing impairment is among the most prevalent sensory impairments in human beings. As documented in various studies, the worldwide prevalence of hearing loss is approximately between 4.0% and 18.1% [1,2,3,4,5]. Despite the uncertainty of this estimate, the associated economic and medical burden can be substantial. Hearing loss is also a critical problem because of its adverse outcomes. Because of communication disorders related to hearing loss, social withdrawal, lower self-esteem, and depression can occur [6,7]. The World Health Organization estimated that up to 360 million people in the world have disabling hearing deficiency. Nearly one-third of people aged 65 years or above are affected by hearing deficit [8]. However, approximately 50% of hearing loss cases are preventable, and a considerable amount of the remaining cases are treatable [7]. Most causes of hearing loss can be prohibited, and several can be treated effectively and immediately. Through personalized management planning, other causes that could not be completely nullified can be rehabilitated through various available measures so that patients can better integrate into society [9].

Nevertheless, numerous countries experience practical challenges in preventing and rehabilitating hearing impairment. The first obstacle is the limited medical resources failing to meet patient demands. The diagnostic benchmark for hearing impairment is the standard audiogram. Nevertheless, financial or geographic restrictions can impede prompt audiogram testing [10]. Advanced diagnostic tools are evolving at a time when standard audiometries are rather scarce. Because of limited medical supplies, primary health care providers struggle to perform hearing assessments. Moreover, qualified audiologists are extremely shorthanded in many underdeveloped and developing countries. Self-performed hearing tests by means of mobile devices can be easily and cost-effectively implemented on a large scale and at the same time sustain a uniform qualification criterion [7,11,12,13,14,15,16,17,18]. They are enormously accessible due to the widespread usage of smartphones and the low manpower requirement of qualified medical staff. Moreover, results obtained through such self-performed hearing tests are comparable to those of standard pure-tone audiometry [11,12,13,14,15,16,17,18].

Accordingly, we attempted to develop a cost-effective hearing examination for evaluating an individual’s hearing condition. Smartphone-based hearing tests have been developed as screening and assessment tools to recognize patients with hearing deficits. Hence, our aim was to assess the efficacy of smartphone-based audiometry tests in screening patients with moderate hearing impairment and to validate these tests against standard pure-tone audiometry. We also evaluated and compared the accuracy of smartphone-based air conduction and bone conduction audiometry self-tests with their corresponding standard pure-tone audiometry tests. Secondarily, we aimed to evaluate the effectiveness of smartphone-based air conduction and bone conduction audiometry self-tests for diagnosing conductive hearing loss.

## 2. Materials and Methods

### 2.1. Participant Selection

In total, 103 participants (206 ears in total) who were aged older than 20 years were recruited to the otology outpatient department of Kaohsiung Veterans General Hospital and Shuang Ho Hospital. Patients with existing otorrhea and cognitive impairment were excluded from recruitment. For both ears of each participant, four hearing examinations were performed randomly: a smartphone-based air conduction audiometry self-test, smartphone-based bone conduction audiometry self-test, standard air-conduction pure-tone audiometry, and standard bone conduction pure-tone audiometry. These tests were performed by a board-certificated audiologist in randomized order and in a soundproof room with an average A-weighted ambient noise level of 35 dB during each visit. Informed consent was obtained from all patients.

### 2.2. Study Design

The four hearing tests for all participants were conducted by a board-certificated audiologist in a soundproof room randomly. The four tests were arranged in random order and there was no time lag between tests. We did not calibrate because we aimed to stick to the reality in which calibration may not be readily available even if the patients use the same smartphone and headphone. Mean hearing thresholds were defined at 500, 1000, 2000, and 4000 Hz. For hearing loss with mean hearing thresholds >40 dB, moderate hearing impairment was impressed. We applied the 2 × 2 tables to summarize the statistics in our study. The results were collected for further evaluation and statistical analysis.

#### 2.2.1. Screening Strategies

##### Smartphone-Based Air Conduction Audiometry Self-Test

**Figure 1:**
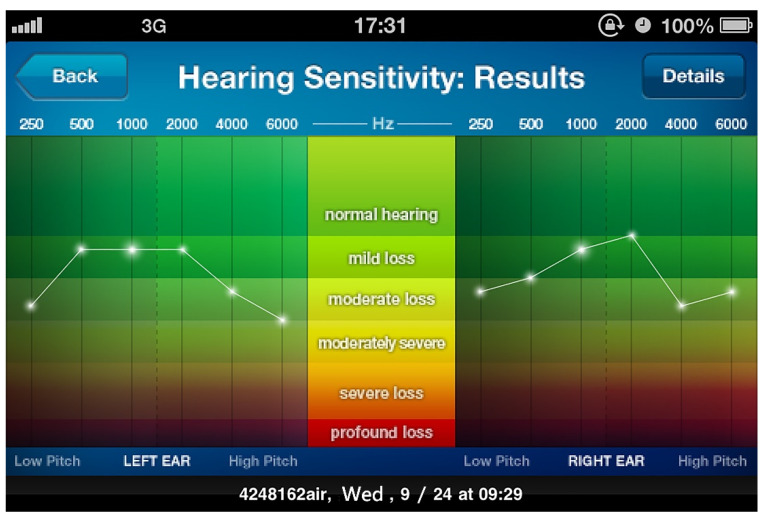
Results of smartphone-based air conduction audiometry self-test for hearing sensitivity, shown in a typical audiogram format.

Audiometry self-tests were completed using an iPhone (Apple Inc., Cupertino, CA, USA). An application named uHear (Unitron, Victoria, BC, Canada), through which patients can test their air conduction pure-tone hearing sensitivity, was downloaded to the phone through the iTunes App Store. uHear employs a “10-dB down and 5-dB up’’ principle [10,12,19], and it can produce calibrated pure tone test stimuli at 250, 500, 1000, 2000, 4000, and 6000 Hz in both ears. The lowest threshold with two positive responses to three transmissions was recorded. An entire hearing test takes only a few minutes. It requires no learning curve to complete the hearing test [10,12,19]. In this study, hearing sensitivity was generated after the hearing tests and it was visualized in a typical audiogram (Figure 1). The smartphone-based air conduction audiometry self-test was conducted in a soundproof room with an average ambient noise level of 35 dB HL or less. Sennheiser hd201 headphones (Sennheiser Electronic Corporation, Lower Saxony, Germany) were applied in the tests. The participants were instructed to tap a large symbol on the smartphone touchscreen when a sound was heard. Prior to the test, a solitary audiologist issued verbal instructions for this smartphone-based air conduction audiometry self-test [10]. All participants completed this smartphone-based self-test in a soundproof room. The results of this self-test and standard air conduction pure-tone audiometry tests were collected for further comparison and evaluation.

##### Smartphone-Based Bone Conduction Audiometry Self-Test

This self-test was also completed with an iPhone (Apple Inc., Cupertino, CA, USA). The uHear application (Unitron., version 1.0, South Africa) was downloaded from the iTunes App Store. The procedure of this smartphone-based bone conduction audiometry self-test was the same as that of the air conduction variant. The results of the hearing sensitivity test were visualized in a typical audiogram format after test completion. Furthermore, the smartphone-based bone conduction audiometry self-test was conducted in a soundproof room whose average ambient noise level was less than 35 dB HL. AfterShokz Sportz 2 bone conduction headphones (AfterShokz Corporation, New York) were used for all participants. This smartphone-based self-test was completed by each participant in a soundproof room. Finally, we put the results of this smartphone-based bone conduction audiometry self-test and those of standard bone conduction pure-tone audiometry tests into comparison.

##### Standard Air Conduction Pure-Tone Audiometry

A Grason-Stadler GSI-61 clinical audiometer (AIC Medical, Oklahoma City, OK, USA) was used to perform the standard pure-tone audiometry test. Telephonics TDH-50P audiometric headphones (Telephonics Corporation, New York, NY, USA) were used to perform the test. All clinical audiometers and accessory devices met 1996 American National Standards Institute S3.6 guidelines [20]. The test was conducted by a board-certificated audiologist in the soundproof room.

##### Standard Bone Conduction Pure-Tone Audiometry

The Grason-Stadler GSI-61 clinical audiometer as well as a B-71 bone conductor (Radioear Corporation, New Eagle, PA, USA) were used to perform the standard bone conduction pure-tone audiometry test. All clinical audiometers and accessories met the 1996 American National Standards Institute S3.6 guidelines [20]. The test was carried out by the board-certificated audiologist in a soundproof room. Degree of hearing loss was evaluated according to the hearing thresholds in each ear of patients.

## 3. Results

In total, 103 patients (a total of 206 ears) were examined in our study. The mean age of the patients was 55.23 ± 6.14 years. Of these patients, 69 were men and 36 were women. Detailed patient demographics are summarized in Table 1. The mean hearing threshold at 500, 1000, 2000, and 4000 Hz in the standard air conduction pure-tone audiometry test was 40.75 ± 14.95 dB. In the standard bone conduction pure-tone audiometry test, the mean hearing threshold at 500, 1000, 2000, and 4000 Hz was 36.47 ± 15.15 dB. The smartphone-based air conduction audiometry self-test and smartphone-based bone conduction audiometry self-test took 3 min on average, respectively. The standard air conduction pure-tone audiometry and standard bone conduction pure-tone audiometry took 6 min, respectively. These four tests were arranged in randomized order. We applied frequencies at 500, 10,000, 2000, and 4000 hz in the audiometry. The average of these frequencies is counted as the hearing threshold. The results of each hearing screening test are listed below.

### 3.1. Standard Air Conduction Pure-Tone Audiometry

Table 1 details the experimental data of the standard air conduction pure-tone audiometry test. The test revealed 88 ears with moderate hearing impairment (pure-tone average PTA > 40 dB HL).

### 3.2. Standard Bone Conduction Pure-Tone Audiometry

Table 1 presents the results obtained from the standard bone conduction pure-tone audiometry test. The test identified 76 ears with moderate hearing impairment (PTA > 40 dB HL).

### 3.3. Smartphone-Based Air Conduction Audiometry Self-Test

As indicated in Table 2, the results obtained from the smartphone-based air conduction audiometry self-test were compared with those from the standard air conduction pure-tone audiometry test. Among the 88 ears identified as having moderate hearing impairment (PTA > 40 dB HL) by the standard air conduction pure-tone audiometry test, 71 registered a PTA of more than 40 dB in the air conduction audiometry self-test conducted with a smartphone, demonstrating 80% sensitivity (95% confidence interval CI = 0.71–0.88). Moreover, among the 118 ears with no evidence of moderate hearing impairment (PTA ≤ 40 dB HL) as documented in the standard air conduction pure-tone audiometry test, 99 registered a PTA of less than 40 dB HL in the smartphone-based air conduction audiometry self-test, indicating 84% specificity (95% CI = 0.76–0.90). Our data revealed that the positive likelihood ratio was 5.01 (95% CI = 3.28–7.66) and that the negative likelihood ratio was 0.23 (95% CI = 0.15–0.36).

### 3.4. Smartphone-Based Bone Conduction Audiometry Self-Test

The results of the smartphone-based bone conduction audiometry self-test were compared with that of the standard bone conduction pure-tone audiometry test, as presented in Table 3. Among the 76 ears with moderate hearing impairment (PTA > 40 dB HL) documented in the standard bone conduction pure-tone audiometry test, 49 recorded a PTA of >40 dB HL in the smartphone-based bone conduction audiometry self-test, indicating 64% sensitivity (95% CI = 0.53–0.75). Furthermore, among the 130 ears not identified as having moderate hearing impairment (PTA ≤ 40 dB HL) in the standard bone conduction pure-tone audiometry test, 92 recorded a PTA of ≤40 dB HL in the smartphone-based bone conduction audiometry self-test, demonstrating 71% specificity (95% CI = 0.62–0.78). Our data revealed that the positive likelihood ratio was 2.21 (95% CI = 1.61–3.02) and that the negative likelihood ratio was 0.50 (95% CI = 0.36–0.69).

Conductive hearing loss is defined as an air–bone gap of at least 20 dB compared with a normal bone conduction hearing threshold. Among the 206 ears, 24 were diagnosed with conductive hearing loss by using the standard air conduction and bone conduction pure-tone audiometry tests. The smartphone-based air conduction and bone conduction audiometry self-tests correctly diagnosed 17 of those 24 ears with conductive hearing loss.

## 4. Discussion

Masalski et al. reported a 15.6% worldwide prevalence of hearing impairment on the basis of 116,733 hearing tests performed by Android users on mobile devices [7,11,12,13,14,15,16,17,18,21]. People in low-income countries account for 80% of the global population with hearing impairment. However, because these countries struggle to provide citizens with even basic medical services to avert other life-threatening diseases, audiology services are overlooked [12]. The demand for audiology services in developing countries has surged because many citizens lack access to hearing health care delivered by audiologists. Audiology services are unequally distributed across the world [12]. Thus, developing fast, easy-to-use, low-cost, and reliable hearing screening methods is crucial.

In our study, we compared results from a smartphone-based air conduction audiometry self-test and those from a standard air conduction pure-tone audiometry test. Among the 88 ears determined as having moderate hearing impairment (PTA > 40 dB HL) by the standard air conduction pure-tone audiometry test, 71 recorded a PTA of >40 dB HL in the smartphone-based air conduction audiometry self-test, indicating an 80% sensitivity. Moreover, among the 118 ears without moderate hearing impairment (PTA ≤ 40 dB HL) documented in the standard air conduction pure-tone audiometry test, 99 registered a PTA of ≤40 dB in the smartphone-based air conduction audiometry self-test, demonstrating 84% specificity. These findings coincide with those of previous studies. Li et al. observed that the sensitivity and specificity of smartphone-based self-tests performed by older (>65 years) adults were 92% and 76%, respectively [12]. Sara et al. reported that a smartphone-based hearing assessment application had 100% sensitivity and 60% specificity (compared with an audiometer) in screening for moderate hearing impairment [22]. In our study, the smartphone-based air conduction audiometry self-test exhibited a sensitivity of 80% and specificity of 84% (compared with standard air conduction pure-tone audiometry) in screening for moderate hearing impairment. Moreover, the mean thresholds at each tested frequency in the smartphone-based air conduction audiometry self-test were higher (which is associated with worse hearing) than those recorded in the standard air conduction pure-tone audiometry test. Our results thus accord with those of related studies. One possible reason for our finding is that the smartphone-based air conduction audiometry self-test demonstrated higher sensitivity, which may lead to overestimations of hearing impairment severity.

Our pioneering study also correlated the accuracy of the smartphone-based bone conduction audiometry self-test and that of the standard bone conduction pure-tone audiometry test. Among the 76 ears with moderate hearing impairment (PTA > 40 dB HL) documented in the standard bone conduction pure-tone audiometry test, 49 registered a PTA of >40 dB HL in the smartphone-based bone conduction audiometry self-test, indicating a sensitivity of 64%. Furthermore, among the 130 ears deemed not to have moderate hearing impairment (PTA ≤ 40 dB HL) in the standard bone conduction pure-tone audiometry test, 92 recorded a PTA of ≤40 dB in the smartphone-based bone conduction audiometry self-test, suggesting a 71% specificity. Previous studies have mostly focused on smartphone-based air conduction audiometry self-test accuracy. By contrast, our study is the first to use AfterShokz Sportz 2 bone conduction headphones to perform experiments on the accuracy of smartphone-based bone conduction audiometry. Moreover, by comparing the results of standard air conduction pure-tone audiometry with those of standard bone conduction pure-tone audiometry, we observed that standard air conduction pure-tone audiometry possessed higher sensitivity as well as higher specificity. This difference was due to the sound leakage engendered by the AfterShokz Sportz 2 bone conduction headphones used in the smartphone-based bone conduction audiometry test. To resolve this problem, we thoroughly compared the AfterShokz Sportz 2 bone conduction headphones with the Sennheiser hd201 headphones, discovering that the Sennheiser hd201 headphones fit snugly in the ear canals. This may explain the higher sensitivity and specificity observed in the smartphone-based air conduction audiometry self-test.

Conductive hearing loss is defined as an air–bone gap of at least 20 dB compared with a normal bone conduction hearing threshold. Among the 206 ears, 24 were diagnosed with conductive hearing loss in the standard air conduction pure-tone audiometry test and standard bone conduction pure-tone audiometry test. Our results indicate that smartphone-based air conduction and bone conduction audiometry self-tests correctly diagnosed 17 of these 24 ears with conductive hearing loss.

The reason why this study is important and novel is that we aimed to develop an accurate method to detect hearing impairment with the use of commonly available portable mobile devices, so that early diagnosis and prompt treatment of hearing impairment can be achieved.

### Limitations

This study has several limitations. First, the standard air conduction and bone conduction pure-tone audiometry protocol requires audiologists to apply masking, which aims to acoustically separate the two ears. However, when conducting the smartphone-based air conduction and bone conduction audiometry self-tests, we did not apply masking noise. During the hearing tests, the contralateral ear may hear a tone presented in the tested ear. This phenomenon is called crossover, and it occurs when an air-conducted signal is intense enough to cause the skull to vibrate. Sound is transmitted through bone conduction, allowing the patient to hear the tone in the nontested ear. In future studies, changes should be introduced to the application to prevent improper reaction to the masking noise.

Second, we used AfterShokz headphones in our study because it is one of the most common bone conduction earphones available in Taiwan. We could hardly find mastoid bone-conduction earphones. Despite the fact that the AfterShokz headphone has its bone conduction through the temporomandibular joint, we chose it as the experimental device because of its widespread popularity in the real world. We did not calibrate because we aimed to stick to the reality in which calibration may not be readily available even if the patients use the same smartphone and headphone. We performed the experiment in a soundproof booth in order to minimize the uncontrollable sound effect of the surrounding environment. We aimed to investigate the efficacy of the smartphone-based air audiometry self-test under the circumstance with standardized controllable factors. Testing in more realistic scenarios will be a direction of further research.

We chose mobile phones instead of laptops because of their higher coverage in many developing countries. Thanks to the production of domestic mobile phone models, mobile phones have become more affordable for people’s daily communication. The overall cost of our study was 330 US dollars, consisting of 200 US dollars for the mobile phone, 50 US dollars for the air conduction earphone, and 80 US dollars for the bone conduction earphone.

Besides, in order to minimize possible errors, all participants underwent the experiment under an audiologist’s guidance. It is agreed that if patients were asked to carry out the experimental procedures by themselves, many of them may fail to perform the test, which in turn causes larger procedural variation and affects the participant inclusion of our study. We included the audiologist’s instruction because many people in Taiwan are not familiar with English and it would be difficult for them to carry out the audiological tests on themselves. In order to deal with these unanswered questions, we hope to carry out further investigation in the future. We wish to perform further studies without the guidance of an audiologist.

## 5. Conclusions

Both smartphone-based air conduction and bone conduction audiometry self-tests demonstrated high sensitivity and high specificity in diagnosing moderate hearing impairment. The mobile health application was a user-friendly personalized mHealth application, which aids precise evaluation and early intervention of moderate hearing impairment. Compared with a standard pure-tone audiometry test, the smartphone-based air conduction and bone conduction audiometry self-tests proved capable of detecting conductive hearing loss with reliable sensitivity and specificity. In conclusion, it is a reliable hearing screening tool but can only be achieved if hardware is correctly calibrated or at least standardized and the examination is carried out in a quiet environment. We will refine the mobile health application based on users’ experience and the collected data

## 6. Patents

We thank all participants and their families for supporting the study.

## Figures and Tables

**Table 1:** Patient demographics.

	Number
Total number	103
Age (years)	
20–40	30
41–60	29
61–80	25
81–100	19
Average Age (y)	55.23 ± 6.14 y
Sex	
Male	69
Female	36
Number of ears recorded through standard air conduction pure-tone audiometry	
≤25 dB	80
26–40 dB	38
41–55 dB	41
56–70 dB	21
71–90 dB	15
≥91 dB	11
Mean threshold from standard air conduction pure-tone audiometry	40.75 ± 14.95 dB
Mean threshold from air conduction smartphone-based audiometry self-test	49.54 ± 14.93 dB
Number of ears recorded through standard bone-conduction pure-tone audiometry	
≤25 dB	88
26–40 dB	42
41–55 dB	33
56–70 dB	19
71–90 dB	15
≥91 dB	9
Mean threshold from standard bone conduction pure-tone audiometry test	36.47 ± 15.15 dB
Mean threshold from smartphone-based bone conduction audiometry self-test	46.85 ± 16.26 dB

**Table 2:** Accuracy of the smartphone-based air conduction audiometry self-test as a screening tool compared with the standard air conduction pure-tone audiometry test upon each ear.

		Standard Air Conduction Pure-Tone Audiometry Test		
		PTA > 40 dB	PTA ≤ 40 dB	Total
Smartphone-based air conduction audiometry self-test	PTA > 40 dB	71	19	90
	PTA ≤ 40 dB	17	99	116
	Total	88	118	206
Sensitivity: 0.80 (95% CI = 0.71–0.88); specificity: 0.84 (95% CI = 0.76–0.90); positive likelihood ratio: 5.01 (95% CI = 3.28–7.66); negative likelihood ratio: 0.23 (95% CI = 0.15–0.36)				

**Table 3:** Accuracy of the smartphone-based bone conduction audiometry self-test as a screening test compared with the standard bone conduction pure-tone audiometry test upon each ear.

		Standard Bone Conduction Pure-Tone Audiometry Test		
		PTA > 40 dB	PTA ≤ 40 dB	Total
Smartphone-based bone conduction audiometry self-test	PTA > 40 dB	49	38	87
	PTA ≤ 40 dB	27	92	119
	Total	76	130	206
Sensitivity: 0.64 (95% CI = 0.53–0.75); specificity: 0.71 (95% CI = 0.62–0.78); positive likelihood ratio: 2.21 (95% CI = 1.61–3.02); negative likelihood ratio: 0.50 (95% CI = 0.36–0.69)				

## Data Availability

The data that support the findings will be available on request under the corresponding author’s e-mail: b101090126@tmu.edu.tw.
